# Smartphone use, gender, and adolescent mental health: Longitudinal evidence from South Korea

**DOI:** 10.1016/j.ssmph.2024.101722

**Published:** 2024-10-22

**Authors:** Robert Rudolf, Najung Kim

**Affiliations:** a College of International Studies, Korea University, 145, Anam-ro, Seongbuk-gu, 02841, Seoul, South Korea; b Graduate School of International Studies, Korea University, 145, Anam-ro, Seongbuk-gu, 02841, Seoul, South Korea

## Abstract

• We use longitudinal data and FE-IV technique to identify causal effects of smartphone use on adolescent mental health.
• We find that longer smartphone use causes increased depressive symptoms and higher suicidal ideation for girls, not for boys.
• Social and active smartphone usage such as communicating with friends and family predicts lower depressive symptoms.
• Personal history of depressive symptoms and the timing of puberty moderate the effect of SUT on depressive symptoms.
• Addictive smartphone behavior is frequent among adolescents, and more prevalent among girls than boys.

## 1 Introduction

Smartphones play a key role in adolescent media use and can be considered a ‘metamedium^1^’ (Humphreys et al., 2018). Modern adolescents often spend many hours per day using their phones to communicate with friends and family, to access video platforms and social media, or to play games (Burns & Gottschalk, 2019). Such activities can be social or non-social active or passive. Depending on the daily extent of smartphone use (quantity) and the type of activities (quality), the devices can have varying effects on adolescent mental health. Since the release of the first iPhone in 2007 there has been a steady increase in the ownership and use of smartphones among adolescents around the globe. At the same time, adolescent mental health problems are on the rise in several countries. These developments have triggered a fast-growing interest of scholars in the effects of digital technology use, in particular smartphones, on the mental health of adolescents (Coyne et al., 2020; Dienlin & Johannes, 2022; Ellis, 2019; Jensen et al., 2019; Keresteš & Štulhofer, 2020; Oh et al., 2024; Park & Lee, 2012). Abi-Jaoude et al. (2020) report that over the last decade, increasing mental distress and treatment for mental health conditions among adolescents in the US and Canada has paralleled a steep rise in the smartphone use by children and adolescents. On the one hand, existing research suggests that the use of digital devices is related to worse mental health (Cha et al., 2023; Dienlin & Johannes, 2020; Twenge et al., 2018; Valkenburg, Meier, & Beyens, 2022). On the other hand, other studies argue that not all types of digital media use have adverse consequences on adolescents' mental health (Marciano et al., 2022; Thorisdottir et al., 2019). However, most studies reviewed reported a correlation between social media use and adolescent mental health rather than addressing the causal direction of the relationship. Dienlin and Johannes (2020) stress that making causal claims about the relationship between smartphone use and mental health is still difficult given the lack of sufficient high-quality studies using large samples and longitudinal designs. Lastly, a few studies found underlying conditions such as less face-to-face interaction (Abi-Jaoude et al., 2020) and initial depressive symptoms (Twenge et al., 2018) that make some adolescents – particularly girls – more vulnerable to negative effects of high smartphone use time (SUT).

South Korea (henceforth “Korea”) has been at the forefront of the digital revolution with one of the highest smartphone penetration rates in the world (Lee et al., 2016). According to recent data, the proportion of adolescents using smartphones for more than 3 h a day is 50 percent in Switzerland, 44 percent in Korea, and 43 percent in the US (Cha et al., 2023). In Korea, the percentage of adolescents using smartphones more than 2 h a day increased notably from 64.3 percent in 2017 to 85.7 percent in 2020 (Lee, 2023). Some studies suggest that the increasing use of smartphones may trigger adverse mental health such as stress, depression and suicidal ideation among Korean youth (Cha et al., 2023; Lee, 2023; Lee & Park, 2023). Research also shows that one-quarter of Korean adolescents have developed smartphone overdependence^2^ (30.0 percent of girls and 21.2 percent of boys) (Lee, 2023). A few recent Korean studies also reveal that problematic smartphone use^3^ (PSU) can disrupt adolescents’ mental health; adolescents with PSU demonstrated a higher risk of anxiety (Lee & Lee, 2023), of depressive symptoms (Kim & Han, 2020) and of suicidal ideation (Lee & Lee, 2023) than the non-PSU group. Within the Korean context, research indicates the presence of predisposing factors, such as smartphone overdependence (Cha et al., 2023; Lee & Lee) and pre-existing depressive symptomatology (Hong, 2022; Lee & Kim, 2022), which render female adolescents particularly susceptible to the adverse effects of SUT.

Given the timeliness of the topic, the objective of the present study is to fill the gap of longitudinal studies investigating the relationship between smartphone use and mental health of adolescents, focusing on four hypotheses: firstly, higher levels of SUT have a negative effect on adolescent mental health; secondly, this relationship may differ based on gender; thirdly, different types of uses have differential effects on mental health; and lastly pre-existing conditions of adolescents may affect the association between SUT and mental health. This study will focus in particular on weekday smartphone use time (SUT) and explore the possibility of the relationship differing by gender. Outcome variables studied include depressive symptoms, suicidal ideation, and life satisfaction. Korea's Children and Youth Panel Survey 2018; KCYPS 2018) offers a unique data set with specific questions on smartphone user behavior, use time, and mental health. Using KCYPS data, we are able to track 2588 students from middle school grade 1 in 2018 to high school grade 1 in 2021. Using the longitudinal nature of the data set, we aim to overcome methodological challenges faced by earlier studies and identify causal effects. This study will further examine potential mechanisms including gender, type of use, addictive behavior, and self-control.

The current study contributes to the existing literature in the following ways: First, by using longitudinal data and a fixed-effects instrumental-variable approach, we can account for two common methodological problems in the literature, i.e. omitted variable bias and reverse causality. This enables us to estimate the causal effect of SUT on adolescent mental health. In contrast, most earlier studies were limited to the analysis of statistical association/correlation. Second, this study highlights gender differences in the nexus between smartphone use and adolescent mental health. Hence, we add to a growing literature that suggests that females may be more dependent on digital technology use and more vulnerable to its detrimental effects. Third, we add to the limited yet significant evidence on different types of digital media use producing differing effects on adolescent well-being. More specifically, we consider various types of smartphone use, in particular social vs. non-social use. Fourth, we contribute to the ongoing debate that some groups may be more vulnerable to the negative effects of smartphone use due to existing sociopsychological problems, e.g. lack of self-control and existing depressive symptoms, as well as the timing of puberty. Lastly, while most studies only focus on adverse outcomes, we include measures of psychological ill-being (depressive symptoms, suicidal ideation) as well as well-being (life satisfaction).

The remainder of this study is organized as follows. Section 2 reviews related literature on smartphone use and mental health. Section 3 introduces data, variables, and methodology used in this study. Section 4 presents our main results and the analysis of potential mechanisms and heterogeneous effects. Finally, section 5 discusses our findings and section 6 concludes the study.

## 2 Review of related literature

The use of smartphones among young adults has increased notably during recent years, motivating more scholarly interest in the effects of smartphone use time (SUT) and user behavior. The effect of SUT on adolescents is of particular interest in many studies given that adolescence is a period where individuals develop mental health through social environment by expanding their social sphere (Keresteš & Štulhofer, 2020; Marciano, 2022; Orben, 2020). Adolescents’ innate tendency for being more social-oriented and sensation-seeking leads to increased smartphone use to access instant messaging and social networking sites which may drive increased use and ownership of smartphones as well as social media use among adolescents due to peer pressure (Kim & Lim, 2021). In this process, due to under-developed cognitive self-control skills, adolescents are less capable of controlling their SUT hence more vulnerable to its negative effects (Marciano et al., 2022; Siste et al., 2021). Adolescence is also a period where individual mental health decreases overall, making young people more susceptible to activities that may cause mental ill-being (Dienlin & Johannes, 2020; Gomez et al., 2013; González-Carrasco et al., 2017; Orth et al., 2015).

Systematic reviews and meta-analyses point out a few common challenges with existing individual studies including collapsing all types of digital media use to a single category and using cross-sectional data with relatively small sample sizes that can decrease precision and effect sizes of estimation results (Dienlin & Johannes, 2020; Marciano et al., 2022). These studies also emphasize the importance of considering gender differences and underlying conditions that may make some adolescents more vulnerable to detrimental effects of SUT. These key considerations are addressed by some individual studies, albeit with remaining challenges.

Firstly, a few studies found gender differences in the use of digital services (e.g. online social networks). More specifically, studies show that female adolescents spend more time using online social networks (OSN) than male adolescents (Abi-Jaoude et al., 2020; Barker, 2009; Booker et al., 2018; Keresteš & Štulhofer, 2020). However, only a small number of studies examined how SUT can affect the mental health of girls and boys differently (Limone & Toto, 2022; Thorisdottir et al., 2019; Twenge et al., 2018) although systematic reviews and meta-analyses point out the importance of this analysis given girls’ vulnerability to negative effects of exposure to digital technology (Abi-Jaoude et al., 2020; Keresteš & Štulhofer, 2020).

Secondly, studies show that effects of digital media use differ depending on the types of use, categorizing the use between active and passive^4^ (Abi-Jaoude et al., 2020; Dienlin & Johannes, 2020; Frison & Eggermont, 2016) or between social and non-social^5^ (Dienlin & Johannes, 2020; Woo et al., 2021). Abi-Jaoude et al. (2020) and Frison and Eggermont (2016) found that active use has a positive impact on adolescent mood, while passive use is more strongly related to negative impact on mood (Abi-Jaoude et al., 2020; Dienlin & Johannes, 2020) and depressive symptoms (Escobar-Viera et al., 2018). Woo et al. (2021) posits that social smartphone use for less than 4 h does not increase the risk of mental health problems, even though the SUT exceeded the conventional screen time guideline of 2 h a day. On the other hand, as for using smartphones for non-social purposes, mental health variables deteriorated when the SUT was 2 h or more, suggesting that using smartphones for social purposes may not be detrimental for adolescent mental health. However, Woo et al. (2021) were limited by the use of a single cross-section of the Korea Youth Risk Behavior Web-based Survey (KYRBS), and thus their study was unable to address issues of reverse causality between SUT and mental health.

Thorisdottir et al. (2019) uses the distinction of types of use between active social and passive social. The authors argue that active social media use tends to be negatively associated with emotional distress after controlling for family structure, relative deprivation, parental support, and time spent on social media, while passive social media use was found to be positively related to anxiety and depressive symptoms (Thorisdottir et al., 2019). However, due to a lack of appropriate data, there is still a limited number of studies that is able to distinguish between different types of use (Dienlin & Johannes, 2020; Thorisdottir et al., 2019), in particular between social and non-social use.

Lastly, studies indicate that underlying conditions such as depressive symptoms, smartphone overdependence and PSU can moderate the effect of SUT on adolescent mental health. Firstly, Hong (2022) and Lee and Kim (2022) posit that adolescents with pre-existing depressive symptoms are more likely to demonstrate higher SUT, particularly in case of girls (Lee & Kim, 2022). Secondly, both smartphone overdependence and PSU are caused by excessive and compulsive smartphone use owing to reduction or loss of self-control. Self-control^6^ can help individuals to cope with frustrated or unsatisfied needs in a way that does not harm their well-being (Schneider et al., 2022). Compromised self-control over smartphone use can produce concerning implications as higher levels of screen time due to inadequate self-control can lead to negative mental health (Cha et al., 2023; Kim & Han, 2020; Oh et al., 2024).

In terms of terminology, according to systematic reviews and meta-analyses, studies used digital technology use including various digital devices (e.g. most notably “smartphones”) (Dienlin & Johannes, 2020) and digital media use (Marciano et al., 2022) as independent variables. Although the focus may be on either the device or service, studies recognize that digital technology use among adolescents takes place *mainly* on smartphones and that adolescents use digital devices primarily for the use of social media. The current study, therefore, focuses on the use of smartphones as the independent variable. With regards to the dependent variable, systematic reviews and meta-analyses recognize that mental health includes concepts of well-being which refers to positive mental health (e.g. life satisfaction, happiness) and ill-being meaning negative mental health (e.g. depression, anxiety, suicidal symptoms, stress, etc.). The current study focuses mainly on ill-being (depressive symptoms and suicidal ideation) although it also includes one well-being indicator (life satisfaction).

## 3 Material and methods

### 3.1 Data

In this study we use four waves of data (2018, 2019, 2020, 2021) from the Korea Child and Youth Panel Survey 2018; KCYPS 2018), a nationally representative longitudinal study with the purpose of establishing data to view complex changes regarding growth and development of children and youth in systemic and multi-dimensional perspectives. Data was collected by the National Youth Policy Institute. KCYPS started in 2018 and follows two cohorts of students on an annual basis: 1.) children that were in 4th grade of elementary school in 2018, and 2.) adolescents that were in 1st grade of middle school in 2018. KCYPS initially targeted 2500 students in each of the two cohorts. The Ministry of Education's 2017 Basic Education Statistics was used as the sampling frame. The sample was extracted using multi-stage stratified cluster sampling (National Youth Policy Institute, 2023). In total, 5197 individuals were surveyed in 2018. The study collects information from children/adolescents and their guardians regarding personal development, their development environment and background parameters of parents. Individual interviews were conducted. For this study, we use data from the middle school cohort. Our main analysis uses a sample of 1185 female and 1403 male students followed from first year of middle school to first year of high school. Attrition was relatively moderate, 94.1 percent (89.3 percent) of students were successfully re-interviewed in the 2nd wave (4th wave).

### 3.2 Key variables

In this study, we employ three measures of adolescent mental health, two measures of ill-being (depressive symptoms, suicidal ideation), and one measure of well-being (life satisfaction).

(1) Depressive symptoms are measured using a 10-item depression scale developed by Kim et al., 1984, Kim et al., 2009. The ten items can be seen in detail in Table A1 in the appendix. Agreement to each item is recorded using a 4-item Likert scale (“strongly disagree” = ‘1’, “disagree” = ‘2’, agree” = ‘3’, “strongly agree” = ‘4’). Cronbach's alpha for the 10 items is 0.915, indicating strong internal consistency of the measure. We take the average value across all ten items to construct an index of depressive symptoms.
(2) Suicidal ideation is the level of agreement to the item “I'm having thoughts of suicide”^7^, one of the items within the 10-item depression scale.
(3) Respondents' self-reported overall life evaluation (henceforth *life satisfaction*) is measured using the level of agreement to the survey item “How do you feel about your current life? - I am satisfied with my life.” Despite being a one-item measure, life satisfaction has been frequently used as a measure of psychological well-being by researchers in the fields of education, psychology, sociology, and economics (Diener, 1994; OECD, 2013). Park (2004) concludes that adolescent life satisfaction is a comprehensive measure of adolescent psychological well-being.

The main independent variable in this study is smartphone use time (SUT). Students were asked to report the time spent using a smartphone on an average weekday and an average weekend day during the last school term. Seven response categories range from “not at all” to “more than 4 h”. For some of the below analysis, we converted the categories to hours, e.g. “not at all” to 0 h, “1 h to 2 h” to 1.5 h, and “more than 4 h” to 4.5 h. This study focuses on smartphone use during weekdays, as those are the days that students are most busy during the school term.

Other control variables used in our main analysis include gender, self-reported health, relations with friends, self-reported academic performance, after-school study hours, exercise hours, household income, location of residence (rural/urban; region) and year dummies. Details about the construction of each variable as well as basic descriptive statistics can be found in Table A1 in the appendix.

Table 1 explores differences in the characteristics of female and male respondents. Female students report significantly higher levels of depressive symptoms and suicidal ideation, as well as a significantly lower level of life satisfaction. As for SUT on an average weekday during the school term, female use (2.16h) was significantly higher than that of males (1.98h). Fourteen percent of females spent more than 4 h using their phones, while this figure was only nine percent for males. As for other time use, female students reported longer after-school study hours but less exercise hours compared to their male counterparts.

**Table 1:** Variable means by gender.

	Female	Male	Diff	P value
*Outcomes*				
Depressive symptoms	1.87	1.72	0.15	0.000
Suicidal ideation	1.61	1.48	0.13	0.000
Life satisfaction	2.90	2.98	−0.08	0.000
*Smartphone use time*				
Not at all	0.02	0.03	−0.01	0.001
<30min	0.05	0.05	−0.01	0.092
30min to 1h	0.17	0.16	0.01	0.509
1h–2h	0.29	0.33	−0.04	0.000
2h–3h	0.23	0.24	−0.01	0.114
3h–4h	0.11	0.10	0.01	0.026
more than 4h	0.14	0.09	0.05	0.000
Smartphone hours	2.16	1.98	0.18	0.000
Own smartphone	0.97	0.97	0.00	0.601
*Control variables*				
Health	3.22	3.29	−0.07	0.000
Relation with friends	3.12	3.04	0.09	0.000
Academic performance	3.29	3.29	0.00	0.872
After school study hours	12.46	12.07	0.39	0.000
Exercise hours	2.15	3.03	−0.88	0.000
HH income	519.20	540.10	−20.90	0.000
Metropolitan city	0.41	0.44	−0.03	0.002
Small & medium cities	0.43	0.42	0.01	0.216
Rural	0.16	0.14	0.02	0.012
*Type of smartphone use (share reported “often")*				
Family call	0.53	0.48	0.04	0.000
Family messaging	0.46	0.38	0.08	0.000
Friends call	0.57	0.48	0.09	0.000
Friends messaging	0.77	0.67	0.11	0.000
SNS (e.g. twitter, Facebook, Instagram)	0.54	0.39	0.15	0.000
Gaming	0.20	0.49	−0.29	0.000
Taking pictures or videos	0.46	0.20	0.27	0.000
Watching TV or videos (e.g. YouTube)	0.60	0.66	−0.05	0.000
Listening to music	0.66	0.55	0.11	0.000
Information search	0.43	0.36	0.06	0.000
Reading (webtoon, e-book)	0.39	0.31	0.08	0.000
Studying or working	0.27	0.19	0.07	0.000

Notes: T-test for differences in means. Female sample N = 4,366, male sample N = 5090. Table shows the descriptive statistics for the samples pooled over time (person-years observations).

### 3.3 Methodology

Given past evidence, we hypothesize that higher levels of SUT have a negative effect on adolescent mental health (H1), but that this relationship may differ based on gender (H2). Moreover, we expect that different types of uses have differential effects on mental health (H3) and that pre-existing conditions of adolescents may affect the association between SUT and mental health (H4).

To test the above hypotheses, we can model mental health of student *i* at time *t*, $mh_{it}$, as follows:

(1) $$mh_{it}=\alpha +\beta sut_{it}+X_{it}^{\prime }\lambda +{R_{it}}^{\prime }\Omega +Yea{r_{t}}^{\prime }\pi +u_{it}$$

where $sut_{it}$ is the average weekday time spent on the smartphone of student *i* in year *t*. The model further includes a vector of control variables, $X_{it}$, as well as region effects ($R_{it}$) and year effects ($Y_{it}$).,^8^^,^^9^

Many past studies have estimated equations similar to the one above, yet often being confined to the use of cross-sectional data. However, this can result in two problems:

(1) Personality traits which are usually not directly measurable can differ substantially across individuals. This form of unobserved heterogeneity in the error term $u_{it}$ can bias regression estimates if personality traits are correlated with right-hand side (RHS) variables, here in particular with $sut_{it}$. This leads to endogeneity bias if, for example, personality types with a higher predisposition for depressive symptoms tend to spend more time on their smartphones. To address this issue, we can exploit the longitudinal nature of our data and use fixed-effects (FE) estimation (the so-called *within* estimator) to control for time-invariant unobserved heterogeneity in the dependent variable. This allows us to control for personality differences that are stable over time, including underlying mental health conditions. The FE model therefore decomposes the error term $u_{it}$ in equation (1) into an individual-specific fixed term ($\mu_{i}$) and an idiosyncratic error term ($\varepsilon_{it}$):

(2) $$u_{it}=\mu_{i}+\varepsilon_{it}$$

(2) A second problem that might arise with the simple estimation of equation (1) is that of reverse causation arising from an idiosyncratic shock to well-being. If, for example, an adolescent experiences depressive symptoms triggered by a sudden shock (e.g., the passing of a close relative or friend), this could lead to increased time spent on the smartphone, resulting in reverse causation. Reverse causation can lead to estimation bias in equation (1), even if fixed-effects estimation (equation (2)) is applied. To address potential bias from reverse causation, we can apply a fixed-effects instrumental-variables (FE-IV) approach. Instrumental variables estimation uses an external source of variation (an instrumental variable) that is correlated with the problematic regressor but is not itself a determinant of the dependent variable. Using a two-stages least squared approach, IV estimation purges the regressor from endogeneity bias and yields a consistent estimate (Wooldridge, 2010). We use school-peer SUT and individual smartphone ownership as instruments for individual SUT. Intuitively, peers' smartphone use can be expected to be a strong predictor and a valid instrument for individual smartphone use since it influences individual SUT while not being a determinant of individual mental health. Peer SUT is calculated as the average weekday smartphone use time of respondents from the same school (explicitly excluding the focal student).^10^ Peer effects on individual behavior have shown particularly relevant in the context of Confucian East Asian societies (Rudolf, in press). Moreover, individual smartphone ownership affects SUT through access to the device. While students that don't own a smartphone can still spend time using their family members' phones, it can be expected that they will do so much less than if the phone was their own. The validity of the two instruments can be evaluated using statistical tests of the relevance of instruments (first stage F-test of excluded instruments) and the exclusion restriction of instruments (Hansen test) which will be reported in regression tables in the next section.^11^ All above models will be estimated separately for female vs. male adolescents to test for potential gender differences in the SUT-mental health nexus.

As an extension to our main analysis, we will investigate additional mechanisms underlying the effect of smartphone use on adolescent mental health. First, we will examine the type of smartphone use. KCYPS includes questions on the frequency of using smartphones for 12 different activities, including among others calling and messaging friends and family, using social media (SNS), watching videos, gaming, and information search. We will examine how the types of use differ between females and males, and how they are associated with mental health. A focus of our analysis will lie on social vs. non-social uses. Second, we will use initial depressive symptoms and the timing of puberty to test for the effects of pre-existing conditions and developmental stages on the SUT-depression nexus. Third, we use additional survey items on addictive smartphone behavior (3 items) and self-control with regard to smartphone use (2 items). We will examine how these behavioral variables differ between genders and how they are associated with SUT.

## 4 Results

### 4.1 Smartphone use time and adolescent well-being

Table 2 presents the results of estimating equations (1), (2) using basic fixed-effects estimation. Estimated coefficients indicate a positive and statistically significant association of SUT with depressive symptoms for both female and male students. Associations are stronger for female students, particularly when spending more than 3 h daily on the phone. This gender-specific effect is further confirmed for suicidal ideation. SUT is positively and statistically significantly associated with suicidal thoughts for girls, yet not for boys. In contrast, results indicate no significant relationship between SUT and life satisfaction. Among other variables, health, relations with friends, and academic performance are negatively associated with adolescent ill-being and positively associated with well-being. In contrast, longer after-school study hours are associated with increased depressive symptoms and suicidal ideation. FE results can control for individual unobserved heterogeneity such as personality differences that are stable over time. However, it cannot solve the problem of reverse causality, which will be done in the next part.

**Table 2:** Smartphone use and adolescent mental health (FE-regressions).

	(1)	(2)	(3)	(4)	(5)	(6)
	Depressive symptoms		Suicidal ideation		Life satisfaction	
	Female	Male	Female	Male	Female	Male
*Smartphone use time*						
Not at all	Reference	Reference	Reference	Reference	Reference	Reference
<30min	−0.0103	0.120∗∗	0.0475	0.133∗	0.121	0.0414
	(0.0748)	(0.0586)	(0.104)	(0.0779)	(0.0933)	(0.0771)
30min to 1h	0.0451	0.0756	0.0777	0.0564	0.0989	0.0259
	(0.0677)	(0.0528)	(0.0938)	(0.0702)	(0.0845)	(0.0694)
1h–2h	0.0612	0.110∗∗	0.0604	0.0500	0.0425	0.00395
	(0.0665)	(0.0515)	(0.0922)	(0.0684)	(0.0831)	(0.0677)
2h–3h	0.155∗∗	0.139∗∗∗	0.171∗	0.0550	0.0198	−0.0108
	(0.0673)	(0.0525)	(0.0932)	(0.0698)	(0.0840)	(0.0691)
3h–4h	0.260∗∗∗	0.138∗∗	0.267∗∗∗	0.0601	0.00544	0.0476
	(0.0698)	(0.0560)	(0.0966)	(0.0744)	(0.0871)	(0.0736)
more than 4h	0.231∗∗∗	0.128∗∗	0.202∗∗	0.0247	−0.0394	−0.0321
	(0.0700)	(0.0570)	(0.0969)	(0.0757)	(0.0873)	(0.0749)
*Control variables*						
Health	−0.167∗∗∗	−0.149∗∗∗	−0.142∗∗∗	−0.113∗∗∗	0.159∗∗∗	0.132∗∗∗
	(0.0160)	(0.0146)	(0.0222)	(0.0194)	(0.0200)	(0.0192)
Relation with friends	−0.221∗∗∗	−0.247∗∗∗	−0.245∗∗∗	−0.170∗∗∗	0.372∗∗∗	0.312∗∗∗
	(0.0208)	(0.0189)	(0.0287)	(0.0251)	(0.0259)	(0.0248)
Academic performance	−0.114∗∗∗	−0.0445∗∗∗	−0.0897∗∗∗	−0.0166	0.0895∗∗∗	0.0674∗∗∗
	(0.0120)	(0.0104)	(0.0166)	(0.0139)	(0.0149)	(0.0137)
After school study hours	0.00775∗∗∗	0.00548∗∗	0.0145∗∗∗	0.00670∗∗	−0.000150	6.76e-05
	(0.00269)	(0.00242)	(0.00373)	(0.00321)	(0.00336)	(0.00318)
Exercise hours	−0.0188∗∗	−0.00724	−0.0142	−0.0175∗∗	0.0185∗	0.00131
	(0.00762)	(0.00632)	(0.0106)	(0.00840)	(0.00951)	(0.00831)
Ln hh income	−0.0352∗	−0.00594	−0.0537∗	−0.00324	0.0420∗	0.00919
	(0.0204)	(0.0193)	(0.0283)	(0.0256)	(0.0255)	(0.0253)
*Rural/urban*						
Metropolitan city	Reference	Reference	Reference	Reference	Reference	Reference

Small & medium cities	0.125	−0.0640	0.0965	0.0343	0.0167	0.206∗
	(0.0873)	(0.0837)	(0.121)	(0.111)	(0.109)	(0.110)
Rural	0.124	−0.0679	0.103	−0.0716	0.00485	0.204
	(0.104)	(0.0947)	(0.144)	(0.126)	(0.130)	(0.125)
*Year*						
2018	Reference	Reference	Reference	Reference	Reference	Reference

2019	−0.108∗∗∗	−0.0252	−0.160∗∗∗	0.0168	−0.0289	−0.110∗∗∗
	(0.0196)	(0.0177)	(0.0272)	(0.0235)	(0.0245)	(0.0233)
2020	−0.111∗∗∗	0.000792	−0.169∗∗∗	−0.00110	−0.0414	−0.205∗∗∗
	(0.0205)	(0.0188)	(0.0284)	(0.0250)	(0.0256)	(0.0247)
2021	−0.0946∗∗∗	0.00830	−0.111∗∗∗	0.0418	−0.0910∗∗∗	−0.238∗∗∗
	(0.0211)	(0.0193)	(0.0293)	(0.0257)	(0.0264)	(0.0254)
Region dummies	Yes	Yes	Yes	Yes	Yes	Yes
Constant	3.861∗∗∗	3.119∗∗∗	3.847∗∗∗	2.463∗∗∗	0.691∗∗	0.906∗∗∗
	(0.224)	(0.229)	(0.310)	(0.304)	(0.279)	(0.301)
No of observations	4366	5090	4366	5090	4366	5090
No of individuals	1185	1403	1185	1403	1185	1403

Notes: Robust standard errors in parentheses; ∗∗∗p < 0.01, ∗∗p < 0.05, ∗p < 0.1.

Table 3 presents results from FE-IV regressions that control for potential reverse causality and thus allows us to interpret SUT coefficients as causal effects. It is thus our preferred estimation model. In contrast to Table 2 regressions, SUT is now measured as a *continuous* variable, given that instrumentation would otherwise be difficult with a full set of categorical dummies.^12^ In line with results from Table 2, FE-IV estimates confirm strongly gendered effects. Spending longer hours on the smartphone is found to increase depressive symptoms and suicidal ideation among female adolescents, while having no effect on male adolescents. Results even indicate a positive effect of SUT on male adolescents’ life satisfaction, while no significant effect was found for female life satisfaction. Tests on instrument validity show high F-statistics of excluded instruments in first stage regressions indicating that the instruments are strong predictors of SUT. Moreover, Hansen p-values are above 0.1 in all models except for the female life satisfaction model, indicating that instruments pass the exclusion restriction in those models.^13^

**Table 3:** Smartphone use and adolescent mental health (FE-IV regressions).

	(1)	(2)	(3)	(4)	(5)	(6)
	Depressive symptoms		Suicidal ideation		Life satisfaction	
	Female	Male	Female	Male	Female	Male
*Smartphone use time*						
Hours per weekday	0.208∗∗∗	0.00370	0.125∗∗	0.00373	0.0328	0.148∗∗
	(0.0404)	(0.0458)	(0.0490)	(0.0580)	(0.0447)	(0.0673)
*Control variables*						
Health	−0.175∗∗∗	−0.136∗∗∗	−0.164∗∗∗	−0.119∗∗∗	0.191∗∗∗	0.141∗∗∗
	(0.0231)	(0.0196)	(0.0303)	(0.0273)	(0.0273)	(0.0272)
Relation with friends	−0.255∗∗∗	−0.270∗∗∗	−0.277∗∗∗	−0.199∗∗∗	0.365∗∗∗	0.302∗∗∗
	(0.0328)	(0.0276)	(0.0403)	(0.0337)	(0.0383)	(0.0403)
Academic performance	−0.104∗∗∗	−0.0500∗∗∗	−0.0893∗∗∗	−0.0277	0.0833∗∗∗	0.0718∗∗∗
	(0.0174)	(0.0152)	(0.0227)	(0.0198)	(0.0213)	(0.0203)
After school study hours	0.00500	0.00354	0.0146∗∗∗	0.00425	−0.000656	0.000149
	(0.00391)	(0.00336)	(0.00534)	(0.00445)	(0.00467)	(0.00425)
Exercise hours	−0.0129	−0.00486	−0.00674	−0.00865	0.00972	−0.0133
	(0.00991)	(0.00852)	(0.0131)	(0.0114)	(0.0119)	(0.0117)
Ln hh income	−0.0816∗∗∗	0.00427	−0.105∗∗∗	0.0359	0.0269	0.0222
	(0.0276)	(0.0285)	(0.0332)	(0.0396)	(0.0278)	(0.0348)
*Rural/urban*						
Metropolitan city	Reference	Reference	Reference	Reference	Reference	Reference

Small & medium cities	0.176		0.192∗∗		−0.170∗∗∗	
	(0.175)		(0.0953)		(0.0632)	
*Year*						
2018	Reference	Reference	Reference	Reference	Reference	Reference

2019	−0.102∗∗∗	−0.0239	−0.158∗∗∗	0.0113	−0.0225	−0.124∗∗∗
	(0.0212)	(0.0188)	(0.0287)	(0.0241)	(0.0256)	(0.0248)
2020	−0.128∗∗∗	0.00830	−0.181∗∗∗	−0.00544	−0.0571∗∗	−0.264∗∗∗
	(0.0230)	(0.0251)	(0.0304)	(0.0326)	(0.0281)	(0.0369)
Region dummies	Yes	Yes	Yes	Yes	Yes	Yes
No of observations	3241	3781	3241	3781	3241	3781
No of individuals	1130	1327	1130	1327	1130	1327
First stage F-stat	75.28	36.27	75.28	36.27	75.28	36.27
Hansen p value	0.619	0.470	0.722	0.195	0.0110	0.601

Notes: Robust standard errors in parentheses; ∗∗∗p < 0.01, ∗∗p < 0.05, ∗p < 0.1.

### 4.2 Types of use and adolescent well-being

Mental health effects of smartphone use can differ depending on the type of usage. KCYPS questionnaires ask adolescents how often they are active in one of 12 different types of smartphone activities.^14^ Table 1 breaks down the usage by sex and reports the percentage of males and females that reported to engage “often” in a certain activity. Approximately half of all boys and girls use smartphones often for family calls, yet females use it more often for messaging and calling friends. SNS usage of females (54 percent) is significantly higher than that of males (39 percent). Females are also more likely to use smartphones for taking pictures and videos. In contrast, males (49 percent) are much more likely than females (20 percent) to engage in gaming often.

We can include dummy variables for frequent engagement in different types of activities into our regression models. Tables A3 and A4 in the appendix report the regressions including the type of smartphone use for female and male adolescent's depressive symptoms, respectively. Regression estimates for the female sample in Table A3, column (1), suggest that messaging with family and friends as well as family calls are associated with lower depressive symptoms for girls. Those activities can be classified as purely active and social activities. Column (2) examines activities that are on average less social and more passive. None of these 5 types of use is significantly related to depression at a 95 percent significance level. Among the 5 types, only watching TV or videos shows a marginally significant, positive association with depressive symptoms. Column (3) examines types of usage that are related to learning and working. Information search, an active usage, is associated with lower depressive symptoms, while reading webtoons or e-books is associated with higher depressive symptoms. Lastly, column (4) includes all activities at once in the model. While some of the earlier effects are confirmed, less activities are statistically significant. This could in part be due the various activities being correlated with one another. Overall, Table A3 results suggest that, ceteris paribus, active and social smartphone usage is associated with lower depressive symptoms in female adolescents.

Table A4 in the appendix repeats the same analysis for male adolescents. Results from column 1 are very similar to those for the female sample. Depressive symptoms are again negatively and significantly associated with family calls, family messaging, and friends messaging. Similar to girls, boys also show a negative relationship between information search and depressive symptoms. In contrast, activities that are correlated with higher depressive symptoms among males are ‘taking pictures or videos’ and ‘studying or working’, both active activities that can be either social or non-social.

In Table 4, we aim to assess the relationship between social use, smartphone use time (SUT), and depressive symptoms more rigorously. Here, social use is the average value of four dummy variables (family call, family messaging, friends call, and friends messaging) and indicates how often adolescents engage in social/communication activities with friends and family on their smartphones. Three different models were tested: Models in (1) and (2) add social use and the interaction term between SUT and social use to the baseline model. Models (3) and (4) are estimated without the interaction terms, but include a squared term for SUT to account for potential nonlinearities. Models (5) and (6) use FE-IV instead of FE.

**Table 4:** Types of smartphone use and adolescent depressive symptoms.

Dep. Var.: Depressive symptoms	(1)	(2)	(3)	(4)	(5)	(6)
	Female	Male	Female	Male	Female	Male
	FE	FE	FE	FE	FE-IV	FE-IV
*Smartphone use*						
Hours per weekday	0.0526∗∗∗	0.0125	0.0974∗∗∗	0.0530∗∗	0.208∗∗∗	0.0430
	(0.0150)	(0.0123)	(0.0278)	(0.0248)	(0.0433)	(0.0612)
Hours per weekday squared			−0.00663	−0.00724		
			(0.00542)	(0.00501)		
Social use	−0.185∗∗∗	−0.158∗∗∗	−0.147∗∗∗	−0.134∗∗∗	−0.209∗∗∗	−0.179∗∗∗
	(0.0512)	(0.0448)	(0.0294)	(0.0256)	(0.0454)	(0.0419)
Hours per weekday ∗ Social use	0.0206	0.0125				
	(0.0216)	(0.0192)				
No of observations	4310	5019	4310	5019	3191	3711
No of individuals	1183	1400	1183	1400	1124	1313
First stage F-stat					60.73	25.77
Hansen p value					0.22	0.63

Notes: Fixed-effects (FE) and fixed-effects instrumental-variables (FE-IV) estimations. All other control variables as in Table 2, Table 3 models. Standard errors in parentheses; ∗∗∗p < 0.01, ∗∗p < 0.05, ∗p < 0.1.

Table 4 results confirm the findings from Tables A1 and A2 that social and communicational activities are negatively related to depressive symptoms. Results also show that hours per weekday remain positively associated with depressive symptoms, especially for girls. Moreover, estimations show that squared terms for SUT are insignificant. Lastly, interaction terms between SUT and social use are insignificant, indicating that social use, while important in itself, does not moderate the negative association between time spent on the smartphone and adolescent mental health.

### 4.3 Pre-existing conditions and development stage

We carried out further heterogeneity analyses by examining how the relationship between adolescent SUT and depressive symptoms may depend on pre-existing conditions and the timing of puberty. We defined pre-existing conditions as reporting higher than median depressive symptoms in the first wave of interviews in 2018 when adolescents were in middle school grade 1. Table 5, panel A shows that, among females, those with pre-existing conditions had a more than twice as strong effect of SUT on depressive symptoms (coeff: 0.239; se: 0.0576) compared to those with no pre-existing conditions (coeff: 0.108; se: 0.0585). For males, both groups had no significant relationship between SUT and depressive symptoms.

**Table 5:** Further heterogeneity analysis.

*Panel A: by pre-existing depressive condition*	(1)	(2)	(3)	(4)
	Female		Male	
	No pre-existing condition	Pre-existing condition	No pre-existing condition	Pre-existing condition
*Smartphone use*				
Hours per weekday	0.108∗	0.239∗∗∗	−0.0200	0.0616
	(0.0585)	(0.0476)	(0.0503)	(0.0663)
No of observations	1449	1792	2181	1600
No of individuals	506	624	770	557
First stage F-stat	28.66	45.19	22.37	16.68
Hansen p value	0.796	0.299	0.748	0.484
*Panel B: by timing of puberty*	(1)	(2)	(3)	(4)
	Female		Male	
	No early puberty	Early puberty	No early puberty	Early puberty
*Smartphone use*				
Hours per weekday	0.258∗∗∗	0.132∗∗	−0.00155	0.0977
	(0.0561)	(0.0575)	(0.0488)	(0.126)
No of observations	2230	1011	2927	854
No of individuals	783	347	1027	300
First stage F-stat	42.55	34.08	30.73	5.054
Hansen p value	0.867	0.834	0.137	0.509

Notes: Dependent variable is depressive symptoms. Fixed-effects instrumental-variables (FE-IV) estimations. All other control variables as in Table 2, Table 3 models. Robust standard errors in parentheses; ∗∗∗p < 0.01, ∗∗p < 0.05, ∗p < 0.1.

We further tested how the timing of puberty may affect the SUT-depression nexus. Students were asked starting from the second wave (middle school grade 2) in which year they experienced their first menstruation (girls) or their first “wet dreams” (boys). We define early puberty if girls had their first period during grade five of elementary school or earlier (reported by 31.2 percent of all girls in the sample). For boys, early puberty was defined as having had the first ejaculation by grade six (reported by 23 percent of all boys in the sample). Results in Table 5, panel B show that early puberty meant a weaker effect of SUT on depressive symptoms during middle school years for girls. The effect is roughly half the magnitude compared to girls that started their period in grade 6 or even during middle school. Again, effects are not significant for male adolescents.

These findings suggest that pre-existing conditions and the timing of puberty moderate the relationship between SUT and depression.

### 4.4 Addictive behavior, self-control, and smartphone use time

KCYPS 2018 data further asks adolescents detailed questions about their smartphone user behavior, in particular including questions related to smartphone dependence and self-control. Table 6 examines five statements that examine signs of smartphone addiction and self-control related to smartphone use. The first three statements indicate symptoms of smartphone dependence. Fifteen percent of females (11.9 percent of males) agreed or strongly agreed to the statement “If I can't use my smartphone, it feels like I've lost the whole world.” Moreover, 26.3 percent of females (21.3 percent of males) indicated that for them “It would be difficult to bear if I could not use my smartphone”. 16.3 percent of females (13.3 percent of males) feel restless and nervous without their smartphone. In addition, two statements examine the ability to control one's SUT. 34.5 percent of females (28.6 percent of males) state that they tried to reduce their SUT but failed. Moreover, 48.1 percent of females (38.8 percent of males) report having problems to stop when using their phone. It is noteworthy that for all five statements, female respondents are more likely to agree than male respondents. In addition, all five statements are positively and highly statistically associated with actual smartphone use hours. Interestingly, again all associations are stronger for girls than for boys. In summary, roughly one in four adolescents shows signs of smartphone addictive behavior and almost half of all adolescents are having trouble self-controlling their smartphone use time. Moreover, girls show significantly higher levels of addictive behavior and lack of self-control, while showing stronger associations between addictive behaviors and SUT.

**Table 6:** Addictive smartphone behavior and associations with smartphone use time.

	*Girls*		*Boys*	
*Statement*	“Agree” or “strongly agree” (in percent)	Association with smartphone use hours	“Agree” or “strongly agree” (in percent)	Association with smartphone use hours
If I can't use my smartphone, it feels like I've lost the whole world.	15	0.071∗∗∗	11.9	0.044∗∗∗
It would be difficult to bear if I could not use my smartphone.	26.3	0.123∗∗∗	21.3	0.075∗∗∗
I feel restless and nervous without my smartphone.	16.3	0.060∗∗∗	13.3	0.038∗∗∗
I tried to reduce my smartphone use time, but I failed.	34.5	0.184∗∗∗	28.6	0.144∗∗∗
Even though I think I should stop when using my smartphone, I keep going.	48.1	0.204∗∗∗	38.8	0.139∗∗∗

Notes: Associations are estimated beta coefficients from bivariate OLS regressions with smartphone use hours as independent variable. ∗∗∗p < 0.01, ∗∗p < 0.05, ∗p < 0.1.

## 5 Discussion

This study investigates the causal effects of smartphone use time (SUT) on adolescent mental health using nationally representative panel data for South Korea. It reveals gender-specific impacts and the differentiated effects by type of smartphone usage. The study also highlights pre-existing conditions, e.g. existing depressive symptoms and the timing of puberty, which may affect the relationship between SUT and mental health outcomes.

### 5.1 Effect of SUT on adolescent mental health and gender differences

The present study indicates that there is a causal relationship between longer daily smartphone use and increased depressive symptoms and higher suicidal ideation for girls, yet not for boys, in alignment with previous studies. These results corroborate findings by Cha et al. (2023), Hong (2022), Lee and Park (2023) and Twenge et al. (2018) that posit adolescents who spend more time on digital devices like smartphones are more likely to develop mental health problems such as depressive symptoms and suicidal ideation. The gender disparity demonstrated in our study confirms the findings of previous studies (Abi-Jaoude et al., 2020; Limone & Toto, 2022; Thorisdottir et al., 2019; Twenge et al., 2018) who reveal girls' particular vulnerability to the negative impacts of social media/smartphone use. Abi-Jaoude et al. (2020) and Twenge et al. (2018) argue that girls spend more time on social media than boys, exposing them more to potentially negative impacts. According to Thorisdottir et al. (2019), passive social media use in particular was more strongly related to symptoms of depressed mood among girls. Several studies explain girls' vulnerability to negative mental impact of SUT citing girls' longer hours spent on smartphones (Abi-Jaoude et al., 2020; Twenge et al., 2018). Longer hours spent on social media can translate into higher exposure to negative interactions and misinformation that may trigger negative impact. Studies also refer to the role of mediators to explain girls’ vulnerability. Firstly, studies indicate that use of social media platforms is associated with social comparison (e.g. body image, social comparison) which may lead to negative mood and depression (Arias-de la Torre et al., 2020; Fardouly et al., 2015; Holland & Tiggemann, 2016). Secondly, research reveals that girls report higher levels of Fear of Missing Out (FoMO)^15^ (Beyens et al., 2016; Stead & Bibby, 2017) and that individuals more prone to FoMO tend to use social media more often, thus fueling a vicious cycle and leading to an even higher engagement with these platforms (Beyens et al., 2016; Hayran & Anik, 2021; Marciano et al., 2022). The current study is also related to recent studies showing that girls are more vulnerable to external stressors during adolescence such as increasing learning pressure and school-related stress in wealthy nations (Rudolf & Bethmann, 2023; Rudolf & Lee, 2023).

Our findings on the effects of smartphone use on mental health conditions, with particular vulnerability of girls, point to the need for relevant deterrents imposed by educators and parents. Firstly, the efficacy of smartphone bans in schools is hotly debated with the latest PISA results indicating that such bans, although reducing in-class distractions do not entirely prevent student use (OECD, 2023). In addition, smartphone bans may suppress the devices' positive role to provide enhanced learning environments (Limone & Toto, 2022) and to facilitate young people's connection with multiple social circles, reducing their perception of loneliness or isolation (Arias-de la Torre, 2020; Frison & Eggermont, 2016). This calls for more tailored policy intervention that can promote positive use for educational purposes and discourage excessive screen time particularly for girls. Alternatively, efforts can be made to reduce smartphone addiction through parental mediation through which parents try to maximize their children's online opportunities while also minimizing negative risks by restricting exposure (Livingstone et al., 2017). Livingstone et al. (2017) introduces two parental mediation strategies – restrictive mediation and enabling mediation. Restrictive mediation employs rules, time limits, and bans on particular activities using filters and/or monitoring software installed for instant messaging, etc. Although restrictive mediation may be effective in mitigating risks, it can also restrict positive educational use of smartphones, similarly to a smartphone ban. Enabling mediation strategy refers to active mediation through parents engaging with and monitoring children's online activities with a guiding role to promote internet safety. Its aim is to create a safe framework built based on child agency to promote children's positive use of the internet. Research demonstrates that enabling mediation becomes more effective when parents and children have strong digital literacy (Livingstone et al., 2017) and that fostering digital literacy is essential to promote equal well-being outcomes of children (Burns & Gottschalk, 2019), calling for evidence-based and balanced strategies to promote digital literacy among students, educators, and parents alike.

### 5.2 Effects of different types of use

The current study suggests that *social types* of smartphone usage are associated with lower depressive symptoms, yet they do not moderate the adverse effect of SUT on adolescent mental health. The results of the present study confirm findings of Frison and Eggermont (2016), Thorisdottir et al. (2019) and Woo et al. (2021) that demonstrate digital media use for social purposes is related to decreased mental ill-being. The current study also corroborates the findings of Abi-Jaoude et al. (2020) and Frison and Eggermont (2016) that active social media use had positive impact on mood. Two concepts can be employed to explain the positive impact of social media use. Firstly, the protective factors such as self-esteem and peer relationships gained from social use may confer protection against emotional distress (Thorisdottir et al., 2019). Secondly, the effect of social support^16^ was also used by Frison and Eggermont (2016), Arias-de la Torree (2020) and Woo et al. (2021) to explain the positive effect of social use of digital media. As suggested by Orben et al. (2020), a nuanced approach to screen time recommendations is crucial to promote quality digital engagement that can enhance mental health. More specifically, Orben et al. (2020) suggests implementing school-based programs that teach adolescents to distinguish between beneficial and potentially harmful smartphone uses. These programs should focus on developing skills for critical evaluation of online content and fostering healthy digital use. Concurrently, parental involvement plays a crucial role in shaping adolescents' smartphone use by providing resources for parents on how to promote healthy smartphone use (Twenge & Campbell, 2018). Lastly, as suggested by Verduyn et al. (2017), smartphone applications can be developed to promote active engagement and limit passive scrolling.

### 5.3 The effect of pre-existing conditions and development stage

Our study reveals stronger impacts of SUT on depressive symptoms for middle school girls with a history of depressive symptoms and those who might currently be undergoing puberty. These findings imply the potential role of pre-existing conditions and development stages that may cause variations in individuals’ SUT and potentially intensify its negative impact as well as the gender-sensitive effect of SUT on adolescent mental health.

Our findings on the effect of pre-existing depressive symptoms are in line with Hong (2022) and Lee and Kim (2022) who also found that female adolescents with initial depressive symptoms are particularly susceptible to the negative mental effects of SUT. Our results regarding the effect of development stage relates to Choukas-Bradeley et al. (2022) which argues that girls experiencing puberty typically have more negative body image of themselves, potentially making them more vulnerable to the negative impact of social media.

These results highlight the importance of early intervention programs for girls with depressive symptoms as well as the need for digital literacy programs that are sensitive to developmental stages. Such programs could help pre-pubescent girls develop the skills to navigate digital spaces more cautiously and better evaluate online content (Livingstone & Helsper, 2010). In addition, it will be beneficial to develop programs to lower the depression levels of adolescent girls before them become at-risk individuals (Lee & Kim, 2021).

### 5.4 Addictive behavior and self-control

Within our study sample we further confirmed earlier research that addictive smartphone behavior and lack of self-control is frequent among adolescents, and more prevalent among girls (15–26.3%; 34.5–48.1%) than boys (11.9–21.3%; 28.6–38.8%). These findings resonate with a number of previous studies that observed heightened vulnerability among girls to longer screen time and its psychological impacts (Boer et al., 2021; Cha et al., 2023; Lee, 2023; Lee & Lee, 2023; Oh et al., 2024; Pereira et al., 2020). Lee (2023) reveals that more girls (30.0 percent) were dependent on smartphones than boys (21.2 percent) and that smartphone overdependence was influenced by depressive symptoms and loneliness. These results point to a potential vicious cycle where girls with higher exposure to smartphones develop negative mental conditions which may further trigger smartphone overdependence which is evidenced to cause negative mental health (Kim & Han, 2020; Lee & Lee, 2023; Oh et al., 2024). These results are more concerning when considering the long-term effects of smartphone overdependence and depression. Studies show that depression from adolescence can have long-term negative impact on cognitive function, reduced physical fitness, and changes in brain structure (Wacks & Weinstein, 2021) and cause higher likelihood of multiple mental disorders including depression (Lemola et al., 2015; Clayborne et al., 2019) and suicide attempts (Jonsson et al., 2011).

Our findings call for timely intervention programs for girls with smartphone overdependence, such as digital literacy programs to promote healthy technology usage habits, that can be integrated to school-based education. However, it is unlikely that digital literacy training and enabling mediation alone can solve the vast problem of addictive behaviors observed today. Policy response to smartphone addiction also needs to include what Livingstone et al. (2017) call restrictive parental mediation, i.e. clear rules and time limits set and monitored by parents.

### 5.5 Limitations and suggestions for future research

This study is not without its limitations. Although the data used in this study is unique and richer than that used by most previous studies, future improvements in data quality are needed to corroborate this study's findings and to better understand underlying mechanisms.

• This study relied on adolescents' self-reported smartphone use data. More granular and objectively measured data on app usage would be needed in the future to better understand the interaction of various types of use and mental health. Furthermore, more detailed data on social media use is also needed to better understand the role of active vs. passive use. This could potentially be achieved via tracking apps or device usage logs. In addition, access to a variety of digital devices should be considered, not just the time spent on smartphones as in the current data set.
• Future research using high-quality data from other countries is also needed to corroborate and generalize the findings from this study, particularly its strongly gendered effects.
• Moreover, future studies may want to investigate long-term effects of different types of smartphone use across diverse adolescent populations and into early adulthood.
• Another limitation is the potential for social desirability bias in self-reported mental health measures: Incorporation of clinical assessments or multi-informant reports could strengthen the validity of mental health data.
• Lastly, further investigation is needed to explore the specific mechanisms through which smartphone use interacts with pre-existing depressive symptoms and pubertal development. Longitudinal studies would be particularly valuable in elucidating the temporal relationships between these factors and in identifying potential protective factors that could inform intervention strategies. Further longitudinal studies are needed to fully understand the long-term impacts of smartphone addiction of adolescents as they transition into adulthood.

## 6 Conclusion

The present study used unique longitudinal data and fixed-effects instrumental-variables techniques to identify causal effects of smartphone use time on adolescent mental health. Our results indicate that there exists a causal relationship between daily smartphone use time and the risk of experiencing depressive symptoms and suicidal thoughts for Korean girls, yet not for boys. Moreover, social and active use of smartphones such as calling and messaging friends and family are associated with lower levels of depressive symptoms, yet do not moderate the adverse effect of SUT on adolescent mental health. Findings further show that pre-existing conditions and the timing of puberty are among the potential mechanisms through which smartphone use time affects adolescent depression. Our results further indicate that addictive smartphone behavior and lack of self-control related to smartphone use is frequent among Korean adolescents, and more prevalent among girls than boys. Given the unique nature of the data set used, this study contributes to the existing literature both methodologically and theoretically, further advancing the understanding of the smartphone-mental health nexus and its underlying mechanisms.

Our findings signal the importance of targeted interventions and policies addressing adolescent smartphone use, particularly for girls, to set effective limits to smartphone use time, and to promote responsible behavior under guidance. To this end, school-based digital literacy programs are important to help adolescents distinguish between beneficial and potentially harmful types of smartphone use. Additionally, early intervention programs that are sensitive to developmental stages will be crucial for at-risk adolescents with pre-existing conditions such as depressive symptoms and smartphone overdependence. Lastly, given the vast prevalence of addictive smartphone behavior and lack of self-control among adolescents, parents play a crucial role in implementing effective time limits for the use of not only smartphones, but digital devices as a whole.

## CRediT authorship contribution statement

**Robert Rudolf:** Writing – original draft, Methodology, Formal analysis. **Najung Kim:** Writing – original draft, Conceptualization.

## Informed consent

This research uses anonymized data collected by the National Youth Policy Institute which is publicly available for research purposes.

## Code availability

STATA code available on request.

## Ethical statement

The data is openly available survey data. It has been collected by the National Youth Policy Institute of South Korea. That is, by definition it has gone through ethical clearance.

## Funding

Robert Rudolf's research was supported by a 10.13039/501100002642Korea University research grant (K2406511) and a National Research Foundation of Korea grant (2024S1A5A2A03037121).

## Declaration of competing interest

The authors declare that they have no conflict of interest.

## Figures and Tables

**Table A1:** Variable description

Variable	Description	Response scale	Mean	SD
Depressive symptoms	10-item depression index (Q18, items 27–36)	4-item Likert scale (“strongly disagree” = ‘1’, “disagree” = ‘2’, agree” = ‘3’, “strongly agree” = ‘4’); index constructed by averaging over all items	1.79	0.60
	27) I don't have much energy.			
	28) Feeling unhappy, sad, or depressed			
	29) I worry a lot			
	30) I'm having thoughts of suicide			
	31) I'm prone to crying			
	32) When something goes wrong, I often think it's my fault.			
	33) Lonely			
	34) I have no interest or interest in everything.			
	35) The future doesn't seem promising.			
	36) Everything is hard			
Suicidal ideation	Level of agreement to item 30) “I'm having thoughts of suicide."	4-item Likert scale as above	1.54	0.71
Life satisfaction	Level of agreement to question “How do you feel about your current life? - I am satisfied with my life.”	4-item Likert scale as above	2.94	0.70
Smartphone use time	Respondent self-report of the time spent using a smartphone on an average weekday	Not at all; <30min; 30min to 1h; 1h–2h; 2h–3h; 3h–4h; more than 4h.		
Smartphone hours	as above	in hours	2.06	1.26
Own smartphone	Whether adolescent owns a smartphone	1 = yes, 0 = no	0.97	
Health	self-rated health relative to peers	1 = not at all healthy; 2 = rather unhealthy; 3 = rather healthy; 4 = very healthy	3.26	0.63
Relation with friends	8-item index of relations with friends	4-item Likert scale (“strongly disagree” = ‘1’, “disagree” = ‘2’, agree” = ‘3’, “strongly agree” = ‘4’); index constructed by averaging over all items	3.08	0.49
	1) Spend time with friends			
	2) My friends confide in me about upsetting and difficult things.			
	3) I am good at telling my stories to my friends.			
	4) I can tell my secrets to my friends			
	5) My friends help me when I do something.			
	6) My friends like me and follow me well.			
	7) My friends are interested in me			
	8) I have good relationships with my friends.			
Academic performance	Self-rated academic performance across all subjects during past semester.	1 = very bad; 2 = bad; 3 = average; 4 = good; 5 = very good	3.29	0.90
After school study hours	Average after-school study hours including four types of time use: visiting Hakwon, private tutoring, self-study, at-school afternoon programs, internet and TV classes. Each item measured from 1 to 7 (see next column). Combined adding up all five items.	1 = Not at all; 2 = less than 30min; 3 = 30min to 1h; 4 = 1h–2h; 5 = 2h–3h; 6 = 3h–4h; 7 = more than 4h.	12.25	3.65
Exercise hours	Time during past week spent on exercise that made the respondent sweat (self-report)	1 = Not at all; 2 = 1h; 3 = 2h; 4 = 3h; 5 = 4h and more.	2.62	1.42
HH income	Average monthly household income	in 10,000 KRW	530.47	223.96
Metropolitan city	Respondent lives in metropolitan city	1 = yes, 0 = no	0.43	0.49
Small & medium cities	Respondent lives in small or medium city	1 = yes, 0 = no	0.43	0.49
Rural	Respondent lives in rural area	1 = yes, 0 = no	0.15	0.35

Notes: N = 9456.

**Table A2:** Sensitivity analysis – including living with both parents

	(1)	(2)	(3)	(4)	(5)	(6)
	Depressive symptoms		Suicidal ideation		Life satisfaction	
	Female	Male	Female	Male	Female	Male
*Smartphone use time*						
Hours per weekday	0.208∗∗∗	0.00376	0.125∗∗	0.00435	0.0322	0.147∗∗
	(0.0404)	(0.0458)	(0.0490)	(0.0579)	(0.0448)	(0.0672)
Live with both parents	−0.0438	−0.00761	−0.0161	−0.104	−0.167∗	0.164∗∗
(1 = yes)	(0.0806)	(0.0714)	(0.110)	(0.0927)	(0.101)	(0.0813)
Observations	3241	3781	3241	3781	3241	3781
Number of pid	1130	1327	1130	1327	1130	1327
First stage F-stat	75.30	36.39	75.30	36.39	75.30	36.39
Hansen p value	0.634	0.470	0.718	0.196	0.0135	0.594

Notes: FE-IV regressions. Standard errors in parentheses; ∗∗∗p < 0.01, ∗∗p < 0.05, ∗p < 0.1. All other variables as in Table 3.

**Table A3:** Types of smartphone use and female adolescent depressive symptoms

Dep. Variable: Depressive				
	(1)	(2)	(3)	(4)
*Smartphone use time*				
Hours per weekday	0.0656∗∗∗	0.0520∗∗∗	0.0487∗∗∗	0.0521∗∗∗
	(0.00801)	(0.00834)	(0.00950)	(0.00986)
*Type of use (often)*				
Family call	−0.0525∗∗			−0.0166
	(0.0218)			(0.0260)
Family messaging	−0.0430∗∗			−0.0216
	(0.0209)			(0.0250)
Friends call	0.00513			−0.0171
	(0.0205)			(0.0240)
Friends messaging	−0.0563∗∗			−0.107∗∗∗
	(0.0241)			(0.0283)
SNS		0.0333		0.0142
		(0.0206)		(0.0249)
Gaming		0.0289		0.0119
		(0.0234)		(0.0278)
Taking pictures or videos		−0.0167		−0.0186
		(0.0192)		(0.0231)
Watching TV or videos		0.0336∗		0.0244
		(0.0196)		(0.0238)
Listening to music		0.0242		0.0145
		(0.0204)		(0.0250)
Information search			−0.0850∗∗∗	−0.0734∗∗∗
			(0.0222)	(0.0228)
Reading (webtoon, e-book)			0.0598∗∗	0.0561∗∗
			(0.0235)	(0.0238)
Studying or working			0.0217	0.0287
			(0.0246)	(0.0247)
Constant	3.867∗∗∗	3.827∗∗∗	3.399∗∗∗	3.435∗∗∗
	(0.216)	(0.217)	(0.245)	(0.244)
No of observations	4310	4310	3196	3196
No of individuals	1183	1183	1155	1155

Notes: Fixed-effects estimation as in Table 2. All other control variables as in Table 2 models. Female sample only. Standard errors in parentheses; ∗∗∗p < 0.01, ∗∗p < 0.05, ∗p < 0.1.

**Table A4:** Types of smartphone use and male adolescent depressive symptoms

Dep. Variable: Depressive symptoms				
	(1)	(2)	(3)	(4)
*Smartphone use time*				
Hours per weekday	0.0190∗∗	0.0149∗	0.00961	0.0103
	(0.00758)	(0.00771)	(0.00900)	(0.00912)
*Type of use (often)*				
Family call	−0.0396∗∗			−0.0177
	(0.0198)			(0.0241)
Family messaging	−0.0344∗			−0.0486∗∗
	(0.0196)			(0.0236)
Friends call	−0.0140			−0.00666
	(0.0190)			(0.0227)
Friends messaging	−0.0479∗∗			−0.0631∗∗∗
	(0.0199)			(0.0240)
SNS		0.0357∗		0.0440∗
		(0.0187)		(0.0228)
Gaming		−0.0121		−0.0329∗
		(0.0171)		(0.0197)
Taking pictures or videos		0.0973∗∗∗		0.121∗∗∗
		(0.0204)		(0.0240)
Watching TV or videos		−0.0348∗		−0.0150
		(0.0182)		(0.0220)
Listening to music		0.00357		0.0688∗∗∗
		(0.0177)		(0.0212)
Information search			−0.105∗∗∗	−0.113∗∗∗
			(0.0204)	(0.0208)
Reading (webtoon, e-book)			0.0284	0.0207
			(0.0224)	(0.0228)
Studying or working			0.0872∗∗∗	0.0714∗∗∗
			(0.0247)	(0.0246)
Constant	3.415∗∗∗	3.155∗∗∗	3.203∗∗∗	3.399∗∗∗
	(0.226)	(0.226)	(0.226)	(0.228)
No of observations	3713	5019	5019	3713
No of individuals	1347	1400	1400	1347

Notes: Fixed-effects estimation as in Table 2. All other control variables as in Table 2 models. Male sample only. Standard errors in parentheses; ∗∗∗p < 0.01, ∗∗p < 0.05, ∗p < 0.1.

## Data Availability

Data will be made available on request.

## Footnotes

7 In Korean: „죽고 싶은 생각이 든다.“ (Literally: I'm having thoughts that I want to die.).

10 It should be noted that when using the peer instrument, the number of waves used in the estimation is reduced from four to three with the three years in middle school remaining. The reason is that after completing middle school, students move on to different high schools, and consequently the number of schools in the sample increases, while the number of students per school decreases. This prohibits the estimation of peer effects in high school.

11 For the instruments to be valid, the first stage F-stat should be larger than 10, and the Hansen test p-value should be smaller than 0.1.

## Footnotes

1 Metamedium refers to a digital platform on which a variety of different media can be reproduced and recombined (Jensen, 2016).

2 Smartphone overdependence refers to the state where individuals experience problems caused by excessive smartphone use owing to reduced ability to voluntarily control smartphone use time in accordance with the subjective goals (Lee, 2023). The National Information Society Agency (NIA) developed the Smartphone Overdependence Adolescents Scale which is a self-answered questionnaire consisting of 10 items including 3 about “self-control failure,” 3 about “salience,” and 4 about “serious consequences.” Responses are based on a 4-point scale: 1 = “not at all” to 4 = “very true.” The total scores range from to 10–40 and scores exceeding 22 indicate smartphone overdependence in youth (National Information Society Agency, 2023).

3 Problematic smartphone use (PSU) refers to symptoms of compulsive and dependent use that are similar to those of addiction, including loss of control (Lee & Lee, 2023).

4 Active use of digital devices refers to activities such as chatting, posting, gaming, or creating and sharing contents, whereas passive use can be defined as consuming the contents of others.

5 Social use of smartphones means using them for social purposes such as calling, text messaging, chatting, and engaging in social media, while non-social use refers to activities with non-social purposes such as consuming news and entertainment contents, as well as gathering information.

6 Self-control can be defined as the ability to override or change one's inner responses, as well as to interrupt undesired behavioral tendencies and refrain from acting on them (Tangney et al., 2018).

8 It is standard in panel data analysis using national data to include both time and space controls. Adolescent well-being (and the way it is reported) may differ across geographic regions, as well as over time, and some students within the dataset might have moved from one region to another over time.

9 Given that two waves of our data fall into the COVID-19 pandemic, year effects will be able pick up mental health effects caused by the pandemic.

12 The number of instruments needs to be at least as high as the number of endogenous variables in IV estimations. In order to conduct instrument tests (Sargan/Hansen test), it needs to be higher.

13 As a sensitivity check, an anonymous reviewer suggested to include into the model the variable “living with both parents” as a potential time-varying confounder that might affect the results. We check for this in Table A2 which adds the variable to the earlier model in Table 3. The estimates of Table A2 are consistent with those in Table 3 and thus confirm our findings.

14 Possible responses included “never”, “almost never”, “sometimes” and “often”.

15 The term Fear of Missing Out (FoMO) is defined as a pervasive apprehension that others might be having rewarding experiences from which one is absent (Abi-Jaoude et al., 2020).

16 Social support refers to social interaction through which emotional concerns, instrumental aid, or information is perceived (Dunkel Schetter & Brooks, 2009).
